# Classification of Samples via Neural-Network Augmented Two-Dimensional Infrared Spectroscopy

**DOI:** 10.1021/acs.jpcb.4c08573

**Published:** 2025-05-01

**Authors:** Evan B. Schroeder, Christopher M. Cheatum

**Affiliations:** Department of Chemistry, University of Iowa, Iowa City, Iowa 52242, United States

## Abstract

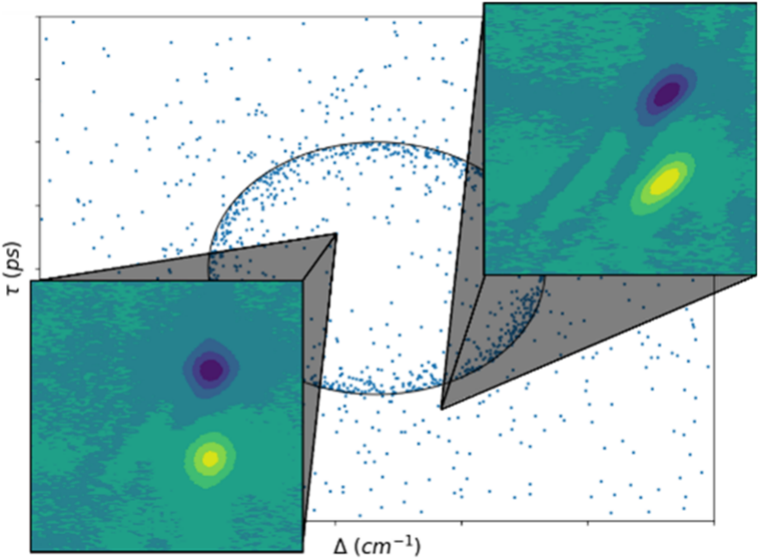

The application of artificial neural network (ANN) techniques to spectroscopy has proven to be a powerful tool for the rapid and accurate classification of experimental samples. However, despite the unique abilities of two-dimensional infrared spectroscopy (2D IR), the use of ANNs to classify samples on the basis of their 2D-IR spectra has been unexplored. We present two investigations into utilizing ANNs to perform end-to-end classification of samples from their 2D-IR spectra. In the first, we construct a model that can perform a binary classification of experimental samples on the basis of their solvent. In the second, we demonstrate that classification is possible even for a single spectral slice of pump-delay and waiting-time combination even when samples display almost identical spectra. These results clearly demonstrate the potential of ANN-augmented 2D IR, with particular emphasis on its use as a technology for high-throughput screening applications.

## 1 Introduction

Two-Dimensional Infrared Spectroscopy (2D IR) is a multidimensional time-resolved pump–probe technique that measures the correlation of vibrational frequencies over time. By exciting a chromophore with a sequence of pulses, 2D IR measures the dynamics of the chromophore’s environment. 2D IR can classify samples by identifying the underlying spectral parameters that are characteristic of the particular sample.^1−5^ However, this analysis often requires large quantities of data and complex analysis.^6,7^ However, in cases where the spectral parameters are not the goal of analysis but instead serve to categorize the state or condition of a system, such vast quantities of data may not be necessary. In cases of binary classification, such as determining if a ligand has bound to a protein, if a mutation affects enzyme active site dynamics, or if a protein is in a specific conformational state, determination of the spectral parameters is but a means to an end. If classification is the ultimate goal, then there is a potential in 2D IR to still achieve accurate results with substantially less data collection.

The need to collect so much data has been necessitated by the means of analysis, chiefly the Center-Line Slope method and Model-Fitting approaches.^7,8^ In either method, the goal is to determine the underlying spectral parameters that define the sample spectra (Section S1). When using the Model-Fitting approach, it is possible to undersample the waiting-time axis (tested to as few as two waiting-times) and still determine these spectral parameters, provided the SNR is large enough; however, even only collecting two waiting-times takes considerably longer than other classification techniques which are considered “high-throughput.”^9−12^

Herein, we investigate the potential of neural-network augmented 2D IR to classify samples with minimal data input. In the case of classifying 2D-IR spectra as either category A or category B, there is some underlying function that takes the spectral data as input and outputs a binary classification score. That function is not obvious and is not trivial to determine. However, by training a neural network based on data where the input and correct output values are known, the weights and biases of the model can be tweaked such that the underlying function is approximated.^13^

Previous work by Rutherford et al. has shown the ability of Machine Learning techniques combined with 2D IR to differentiate protein-drug binding.^4^ These studies serve as an early approach to reduce the dimensionality of 2D IR classification problems and show significant promise of the overall approach, particularly considering the advantages of newer and more powerful neural-network methods. Although others have noted the possibility of utilizing artificial neural networks (ANNs) for 2D-IR spectral classification,^14^ to date, there have been no analyses of the ability of ANNs to classify 2D-IR spectra. Therefore, a proof of concept is needed to demonstrate that neural networks are capable of capturing the patterns present in 2D IR lineshapes. To this purpose, we investigate the ability of ANNs to classify experimental spectra of chromophores in various solvents. We then extend that work to comprehensively investigate the ability of ANNs to classify simulated spectra with relatively small spectral differences and determine the minimum data set necessary for accurate classification and evaluate the effects of differing signal-to-noise ratio data. We show that an appropriately trained ANN is capable of binary classification of 2D IR data even with a signal-to-noise ratio (SNR) as low as 2:1. We then go on to characterize how the classification accuracy varies based on the particular choice of pump-pulse delay and waiting-time. We demonstrate that an appropriately trained ANN is capable of binary classification using a single spectral slice for an optimal pump-delay and waiting-time combination, even for samples with only minute relative spectral differences. This result is a powerful demonstration of the information content in these data and the potential of this approach as an enabling technology for high-throughput screening applications.

## 2 ANN classification of experimental spectra

### 2.1 Experimental Spectra

2D IR instrumentation was described in detail previously.^6,15,16^ Briefly, a commercial Ti:sapphire amplifier generates pulses centered at 800 nm. An optical parametric amplifier produces signal and idler beams that mix in a difference frequency generation stage to output mid-infrared pulses centered at 2155 cm^–1^ with 15 μJ per pulse and a pulse duration of 150 fs. A beam splitter separates pump and probe beams with the pump beam sent to a pulse shaper based on a Ge acousto-optic modulator. After transmission through the sample cell, a sum frequency generation crystal mixes band-narrowed, leftover 800 nm light with the probe beam to upconvert into the visible region for data collection using a commercial spectrometer with a 1024-pixel CMOS line array.

**Figure 1:**
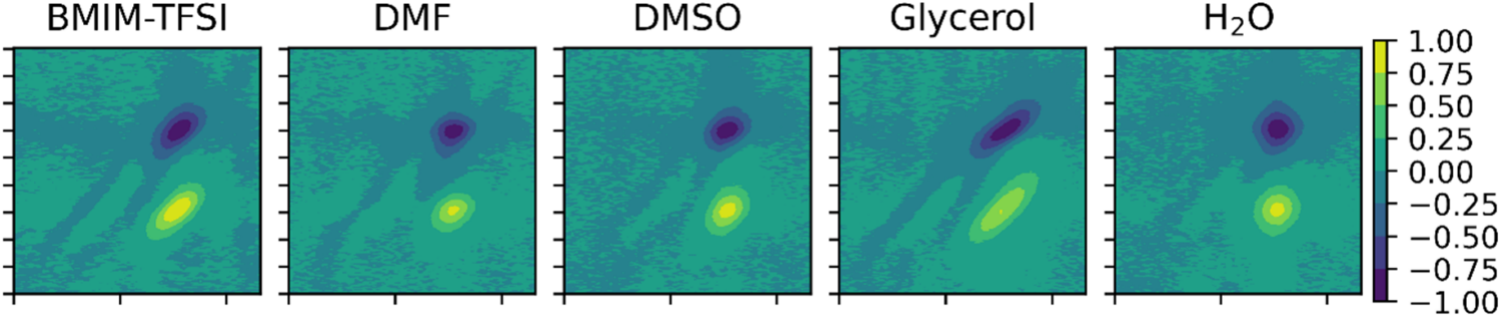
Examples of the SNR 100 data input to the networks for each solvent, post data normalization. Note that since the axis units are arbitrary after normalization, the axis ticks represent intervals of 25 pixels.

We prepare experimental samples of methyl thiocyanate (MeSCN) in varying concentrations in five different solvents: H_2_O, DMSO, DMF, Glycerol, and the ionic liquid BMIM-TFSI. We measure spectra at a single pump–probe delay of 1 ps. All spectra are then normalized prior to their input to the neural network to prevent learning a correlation between a spurious input feature and the desired classification. In brief, the routine consists of cropping, translation, amplitude normalization, and interpolation, followed by the addition of Gaussian noise. A more thorough explanation is given in Sections S2 and S3. Our experimental spectra are collected using an edge-pixel referencing method.^15^ When developing the method, our group determined that after referencing, the background is shot-noise limited, which is modeled using Poisson statistics. However, since we have large counts in these pixels, the Poisson distribution is nearly identical to the Gaussian distribution. As a result, 2D independent Gaussian noise is a realistic approximation of the actual noise in the measurement without experimental artifacts. After the normalization routine, all spectra have identical 0→1 peak pixel positions, frequency ranges, pixel densities, maximum absolute intensities, and signal-to-noise ratio. Thus, the only features available to the neural networks are the line-shape differences inherent to the various samples. Examples of the normalized input data are shown in Figure 1.

### 2.2 Neural-Network Architecture and Training

The objective of the neural networks is to perform a binary classification of some input 2D-IR spectrum, outputting whether the input sample was collected with water as the solvent or not. The 2D IR data input to the networks consists of only a single pump–probe delay, meaning that the data input to the networks are real-valued two-dimensional arrays presented in the frequency-frequency domain.

Of the experimental data collected, roughly half the samples are of MeSCN in H_2_O. In order to maintain a balanced data set, i.e., 50% samples from the positive category and 50% samples from the negative category, additional samples are needed of the H_2_O solvent spectra. To achieve this, additional H_2_O spectra are generated by adding and subtracting Gaussian noise from existing spectra until the number of H_2_O samples yields 50% of the total samples. In total, 54 spectra were generated in this way to achieve a balanced data set. The final data set consists of 1126 spectra, with 100 μ_B_IM-TFSI, 151 DMF, 146 DMSO, 166 Glycerol, and 563 H_2_O.

It should be noted that each spectrum does not necessarily correspond to a unique sample but is, instead, a uniquely collected spectrum. Thus, there is the potential for effective repeats of the same sample, resulting in overfitting of the model. Our approach of adding noise to the spectra to decrease the effective SNR lessens the impact of effective repeats on the training data. For example, consider the Pearson Correlation Coefficient (PCC) of each spectrum relative to a single water solvent sample spectrum. The PCCs of effective repeat spectra do form distinct groups (Figure S3); however, these groups become less distinct at lower SNRs (Figure S4), to the point where at SNR 3 (Figure S5) the effective repeat spectra are no more similar to those within their sample group than they are to spectra outside their sample group. We have additionally found that data sets where the repeats are grouped into a single k-fold and data sets where they are distributed equally among the k-folds produce comparable results (Section S4). As this section is a simple proof of concept of the efficacy of artificial neural networks applied to 2D-IR spectra, a more thorough analysis is appropriate for future studies.

The neural-network architecture is a custom implementation of a ResNet-18 network, a convolutional network designed for two-dimensional image input.^17^ After the final fully connected linear layer of the network, loss is computed via the Cross-Entropy Loss function and the two output classes are rescaled via the Softmax function into pseudoprobabilities so that their collective values sum to one.^18,19^

Training and validation accuracies are calculated using a threshold of 0.5; that is, whichever output class has the higher value after Softmax rescaling is deemed the predicted output class. All training and validation are done with Pytorch, utilizing Pytorch-Lightning as a wrapper.^20,21^ All networks are trained for a maximum of 1000 epochs; that is, the data sets are iterated through the network a total of 1000 times.

When randomly dividing the full data set into training and validation sets, there exists a possibility that the samples of one set might not be representative of the entire data set. This possibility increases when using smaller data sets. To minimize this effect, we use k-fold cross-validation with k = 5.^22^ With five k-folds, the test-validation splits are 80% training to 20% validation.

### 2.3 Results and Discussion

As the classification ability of neural networks applied to 2D-IR spectra is untested, we begin with the identification of samples containing water as a solvent or not based upon their 2D-IR spectra. The differences between samples can be seen by the eye, and so with some training, this is a relatively straightforward task for human vision. We therefore use the experimental spectra classification as a baseline for the potential of neural networks to classify samples on the basis of their 2D-IR spectra.

For networks trained on data presenting signal-to-noise ratios (SNR) of 100, the accuracy of the network when applied to the validation set is 100%; that is, each sample in the validation set is sorted into its binary category correctly. With this result in hand, we add simulated noise to the spectra to test the limits of the networks’ classification abilities. Lowering the SNR of all samples to 50 with the addition of Gaussian noise, we train and validate a new set of networks, also achieving 100% validation accuracy. We continue this for SNRs of 10, 5, 4, 3, 2, 1, and 0, with the validation accuracies presented in Table 1.

**Table 1:** Validation Accuracies Achieved for Each SNR Data Set^a^

SNR	0	1	2	3	4	5	10	50	100
validation accuracy (%)	52.32 ± 0.43	54.89 ± 1.05	86.78 ± 1.31	97.43 ± 0.28	99.56 ± 0.06	100.00 ± 0.00	100.00 ± 0.00	100.00 ± 0.00	100.00 ± 0.00

a Presented are the mean best accuracies of the five k-folds, along with the standard error of the mean.

**Figure 2:**
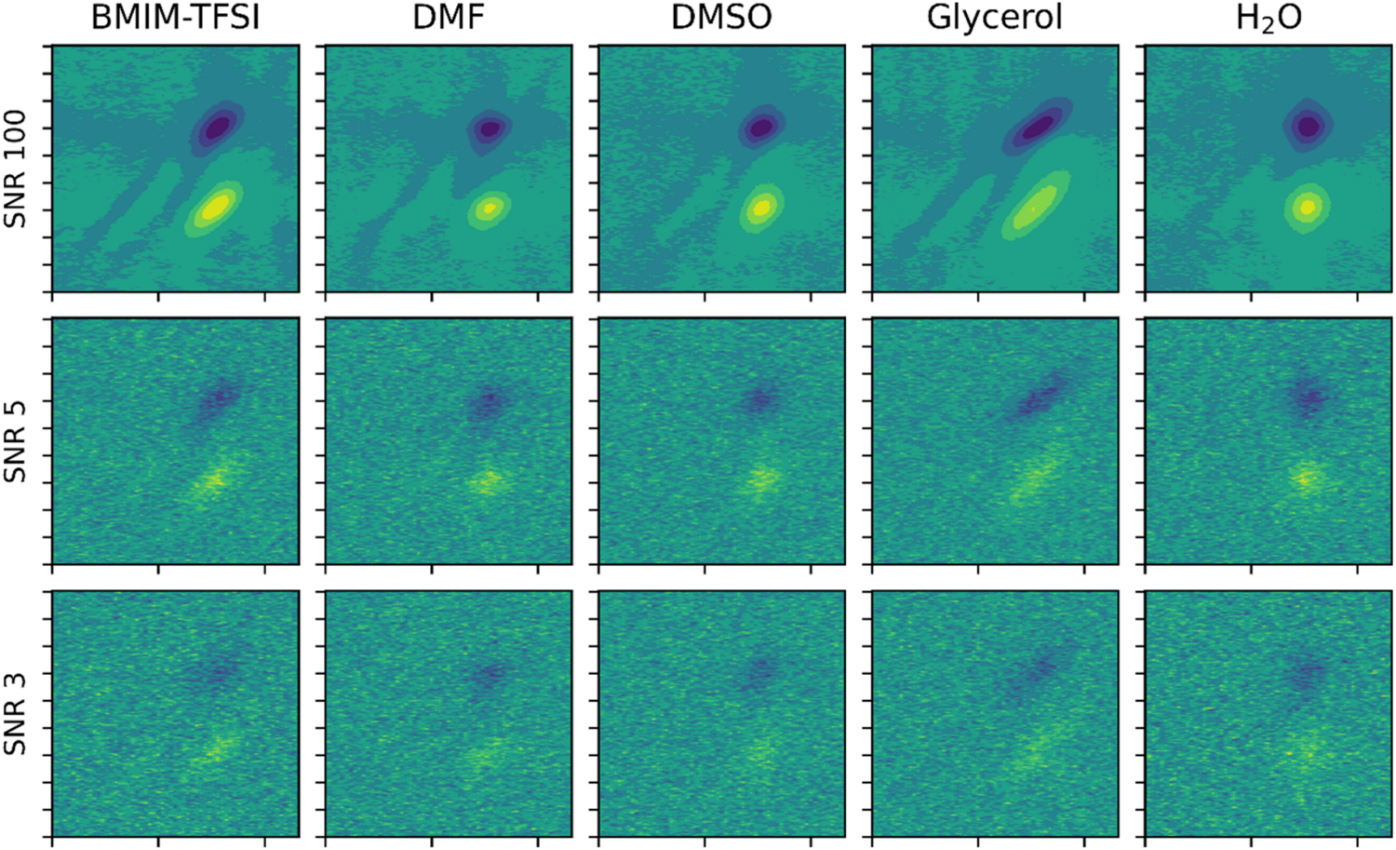
Examples of the normalized data for each solvent as a function of SNR. The models achieved 100% classification accuracy for SNR 100 and SNR 5 data and ∼97% accuracy for SNR 3 data.

While the samples for the SNR 100 data sets are readily distinguishable by the eye, the lower SNR data sets quickly become obfuscated. Figure 2 displays examples of lower SNR samples. Networks perfectly classify all samples of SNR 5 or higher, with near-perfect classification of SNR 4 and 3 samples.

Since the SNR 0 data set is composed of purely Gaussian noise, the networks trained on that data set can serve as a baseline of classification ability, with the SNR 0 data set acting as the reference for no classification ability. An important observation of the SNR 0 results is that the classification accuracy is slightly above 50%. This is most likely due to the accuracy calculation utilizing “binary accuracy” with a threshold of 0.5, whereby whichever output class has the higher value after Softmax rescaling is deemed the predicted output class. By keeping the threshold at 0.5 instead of using a sliding or adaptive threshold, classification results can sometimes become biased toward one of the categories. The loss of each data set indicates the relative difference between the output of the network and the known truth value. By comparing the validation losses of the networks (Figure S6), it is apparent that the networks trained on SNR 1 do display some classification ability, although very little. It is possible that with additional tuning or by training on an expanded data set, classification ability could be improved.

We also analyze the differences between solvents to determine which samples tend to be the “easiest” or “hardest” for the networks to classify. For each SNR, models are trained for 200 epochs, after which the models are frozen and the complete data sets are fed through the network, recording the accuracy and loss for each. Table S1 displays the results of averaging all five k-folds.

For each SNR, we score the solvents by their loss, where the solvent with the highest average loss scores a zero, and the solvent with the lowest average loss scores a four. For example, the order of solvents from best loss to worst loss for the SNR 100 data set is H_2_O, BMIM-TFSI, Glycerol, DMF, and DMSO; therefore, they receive scores of 4, 3, 2, 1, and 0, respectively. We average these scores across all SNRs for each data set, where the averaged scores serve as a metric for how well a specific solvent is categorized relative to others (Table 2).

**Table 2:** Averaged Loss Score for Each Solvent across All SNR Trials, Where a Higher Value Indicates Better Classification Performance Compared to Other Solvents

mean loss score				
BMIM-TFSI	DMF	DMSO	glycerol	H_2_O
3.14	1.14	0.29	2.71	2.71

Across the SNRs, BMIM-TFSI has the best classification score, followed by H_2_O and glycerol tying for second, followed by DMF and DMSO. We expect that the worst categorization ability will occur for those samples on or near the classification boundary; that is, samples with properties not exactly like water but not extremely different. This intuition is reflected in the scoring results. When comparing the differences of the sample’s spectral parameters relative to water, Glycerol and BMIM-TFSI have greater differences from water, whereas DMF and DMSO are more similar to water (Table S2).

## 3 ANN Classification of Simulated Spectra

With the concept that neural networks can decipher the patterns in 2D-IR spectra established, we next probe the limits of binary classification. We focus on two main areas: how similar 2D-IR samples and their associated spectra can be and what is the smallest amount of 2D-IR data that can be collected yet still result in good classification ability?

The time required to collect 2D IR data can often be quite long due to the need to average measurements to compensate for weak chromophores and to collect enough pump-time and pump–probe delay data points to perform the desired analysis such as center-line slope or model-fitting. However, if the collection time is too long, data quality can suffer due to drifting of the optical setup or degradation of the sample. There are cases, however, where executing such a detailed analysis may not be required, such as performing a binary classification of whether a specific sample is the same as or different from a prior base case. A practical application would be in the screening of potential allosteric effectors for a target enzyme in which a chromophore is placed in the active site. The prior would be the enzyme with no allosteric effector, and the classification screen would seek to determine which potential effectors cause a change in the enzyme dynamics indicative of allosteric binding. In these cases, an accurate classification that is sufficiently efficient in terms of the required SNR and data set size could result in decreased data collection times, allowing for high sample throughput.

In this section, we consider simulated spectra so that we can evaluate the limit of the ability of neural networks to classify spectra with only minute difference, use minimal subsets of the 2D IR data for that classification, determine the optimal subset, and assess the lowest SNR at which such classifications would still be feasible. Using simulated spectra, we attempt to reduce the data collection to the smallest possible unit that still allows for binary classification of spectra, where the differences are quantitatively measurable but indiscernible with the naked eye, which provides a rigorous test of this approach.

### 3.1 Simulation of 2D-IR Spectra

Spectra are simulated according to the Kubo line-shape model, utilizing a custom simulation project written in Python (Section S5).^23^ Simulated spectra consist of complex-valued data, to which Gaussian noise is added to achieve a desired signal-to-noise ratio (SNR), followed by amplitude normalization.

**Figure 3:**
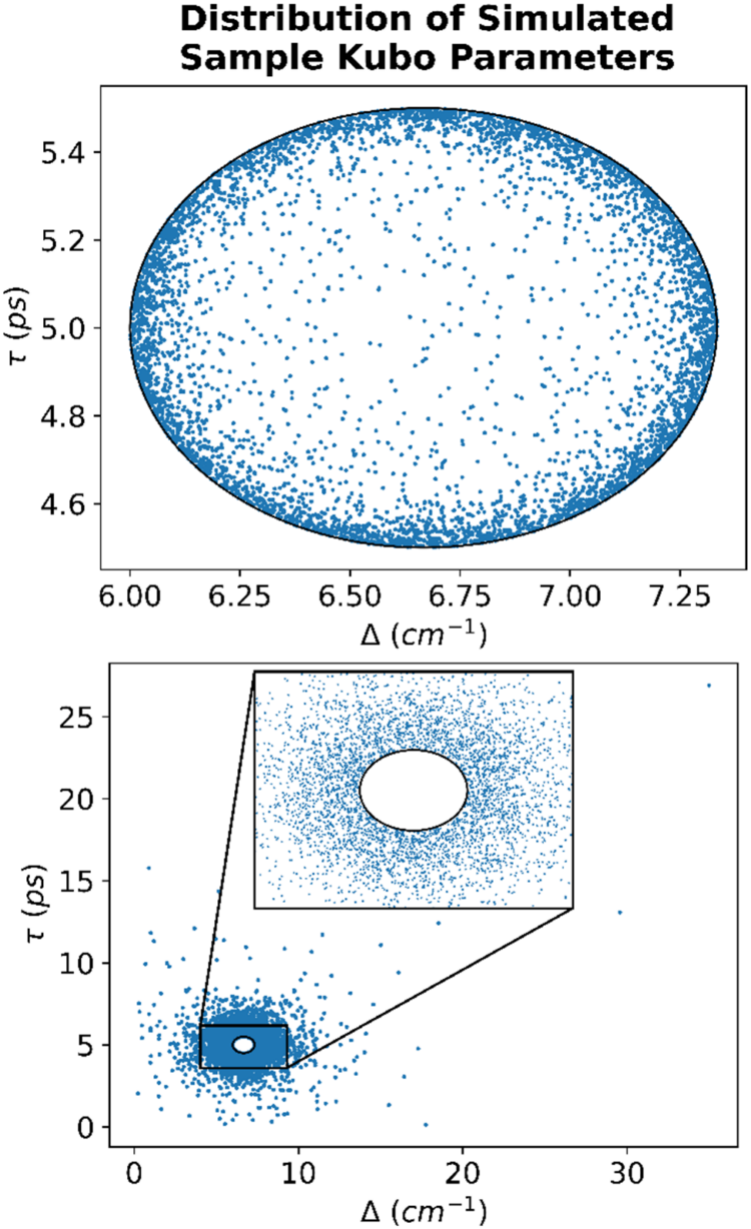
An example of the Kubo parameters for the simulated samples, where the reference values for the data set are (Δ, τ) = (6.7 cm^–1^, 5.0 ps). The upper panel shows the distribution of samples classified as “similar” to the reference sample, and the lower panel shows the distribution of samples classified as “dissimilar” to the reference sample.

The simulated spectra are used to determine the ability of a neural network to distinguish between samples that may have very similar lineshapes. We define “similar” and “dissimilar” according to the percent-different magnitude (PDM) of the Kubo parameters between the two samples. When generating samples, we vary the amplitude (Δ) and time-constant (τ) of a single Kubo component such that the PDM takes the form of eq 1, where the subscript “ref” refers to the Kubo parameters from a reference sample, and the subscript “s” refers to the Kubo parameters for some comparison sample. All other simulation parameters remain constant between the samples, such as 0→1 transition frequency and anharmonicity.
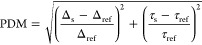
1As an example, consider the reference sample spectral parameters of (Δ_ref_, τ_ref_) = (6.67 cm^–1^, 5 ps), shown as the center of the oval in Figure 3. For a PDM classification boundary of 10%, any samples with a PDM less than or equal to 10% are inside the classification boundary (upper panel) and thus considered “similar” to the reference sample. Alternatively, any samples with a PDM greater than 10% are outside the classification boundary (lower panel) and thus considered dissimilar to the reference sample.

### 3.2 Neural-Network Architecture and Training

For networks trained on simulated spectra, the objective is to perform a binary classification of some input 2D-IR spectrum, as in the networks for experimental spectra. However, the differences between the experimental samples are large enough to be seen by the eye, and so we utilize the simulated spectra to test the discrimination of neural networks applied to 2D-IR samples that display more subtle differences. We therefore use the PDM of samples relative to some reference sample to define the classification boundaries, which allows for the networks to still target binary classification.

For simulated spectra, we utilize a three-dimensional ResNet architecture, which is similar to that used for experimental spectra but which utilizes three-dimensional convolutions instead of two-dimensional convolutions (Section S6). The “channels” input is traditionally used in image classification networks to signify the various RGB channels of an image. However, in this case, the channels input denotes the real or imaginary part of the complex spectrum.

Instead of stopping training for each session at a predetermined maximum number of training epochs, as is done for networks trained on experimental spectra, training is allowed to continue for each session until a custom “early stopping” condition is met, based on the Brier score. The Brier score is a strictly proper scoring-rule like Cross-Entropy Loss, but it is similar to the mean-squared error.^24^ After each training epoch, if the smoothed mean Brier score of the validation set has not decreased in the last 100 epochs and the current gradient of the smoothed mean Brier score is positive, then training is halted.

A total of 10,000 samples are generated for each training session, 50% inside the classification boundary and 50% outside the classification boundary. Due to the large number of samples, k-fold cross-validation is unnecessary for networks trained on simulated spectra. Spectral parameters for each sample are randomly chosen from a one-sided Cauchy distribution, centered at the classification boundary and tailing either inside or outside the boundary depending upon the sample category desired. This is done to ensure that many samples are present at the classification boundary so that the model can learn the relatively minor differences between samples just inside and outside the boundary. Samples far away from the classification boundary (either inside or outside) display significant differences, which the model can more easily classify, and so fewer samples are needed further away from the boundary. An example of the distribution of samples in the parameter space is displayed in Figure 3.

### 3.3 Results and Discussion

In the above case, where networks were designed to classify samples of different solvents, the differences were quite large. Other systems, however, may display only minute differences. We analyze the question of similarity by means of PDM. By defining the classification boundary as 10% PDM relative to a reference sample, we can generate many thousands of samples and probe the ability of the network to discern between similar spectra that display only fractional PDM differences.

As the PDM is defined relative to some reference sample, the choice of reference sample may affect the results. Therefore, we train networks on data sets with reference samples defined with the Kubo amplitude ranging from 3.3 to 13.3 cm^–1^ and the Kubo time-constant ranging from 0.8 to 10.0 ps, totaling 15 different data sets. Each data set contains 10,000 different samples, where 80% are randomly chosen to serve as the training set, with the remaining 20% acting as the validation set, with a new network trained on each data set.

The 2D complex spectrum of a single pump–probe delay contains enough information for the networks to classify samples with near 100% accuracy. Therefore, we reduce the number of pump-times supplied to the network, ultimately using only a single pump-time and a single pump–probe delay. As our experimental 2D-IR setup uses a line array to collect the probe axis, collection of one slice would represent the minimum collection time for our system. Not all slices, however, are equally useful for classification. In 2D-IR spectra, the signal decays as a function of waiting-time. Therefore, choosing a single slice from too large of a waiting-time could result in an input dominated by noise. Samples with similar spectral parameters might not show significant differences at short waiting-times, however. Thus, the selection of a slice that contains the most useful information for classification is not obvious.

We therefore train a series of networks for each data set, with each network trained on a different combination of pump-time and pump–probe delay. For example, for the data set which uses a reference sample defined with Kubo parameters (Δ = 6.7 cm^–1^, τ = 5 ps), we first train a network on data that only contains the slice at pump-time 0 ps and pump–probe delay 0 ps, followed by another network trained on data which only contains the slice at pump-time 0 ps and pump–probe delay 0.05 ps. This continues for a total of 441 networks per data set, with pump-times spanning 0–4 ps and pump–probe delays spanning 0–15 ps. In total across the 15 data sets, we train 6615 separate networks.

We used the Brier Skill Score (BSS) to evaluate the classification ability of each network. A BSS of zero indicates that the model has no better predictive power than random chance; a score of one indicates perfect prediction; and a score less than zero indicates a model with worse predictive power than random chance.

**Figure 4:**
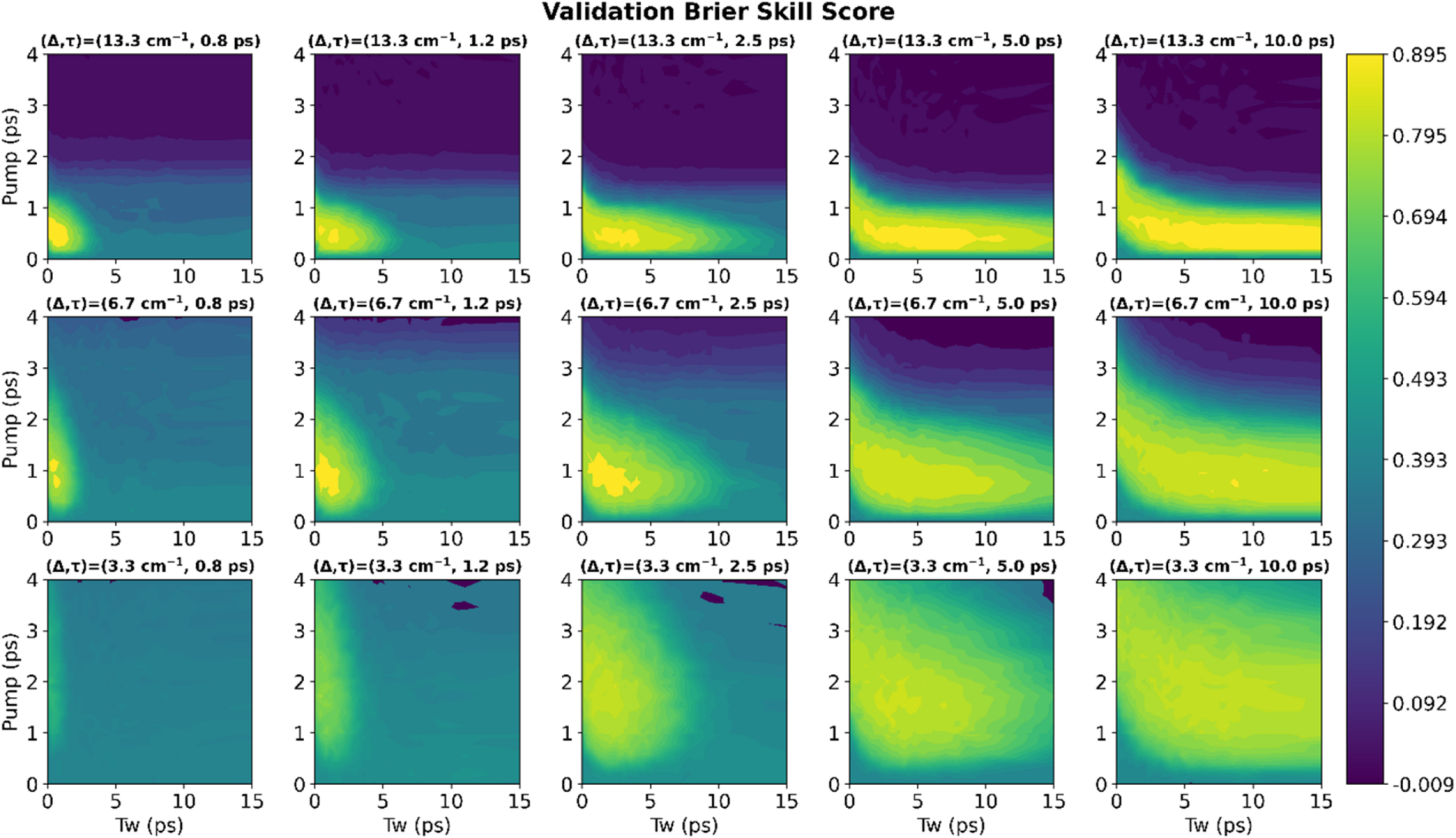
Best BSS achieved for each data set as a function of the pump-time and waiting-time. Note that the data sets are sorted with increasing Kubo amplitude on the exterior *y*-axis and with increasing Kubo time-constant on the exterior *x*-axis.

For each data set, Figure 4 displays the best validation BSS achieved as a function of both the pump-time and the waiting-time of the slice. The contour plots display trends showing which slices tend to be the most informative for classification given the reference Kubo parameters. In general, the optimal pump coordinate is proportional to the Kubo amplitude, and the waiting-time coordinate is proportional to the Kubo time-constant. The maximum validation accuracy obtained for each data set, as displayed in Table S3, is above 90% in all cases except for data set 15. Data set 15 has the smallest Kubo amplitude and smallest Kubo time-constant, which combine to produce a free-induction decay that does not appreciably decay over the pump axis but vanishes extremely quickly across the waiting-time axis, so a low accuracy is not unsurprising.

**Figure 5:**
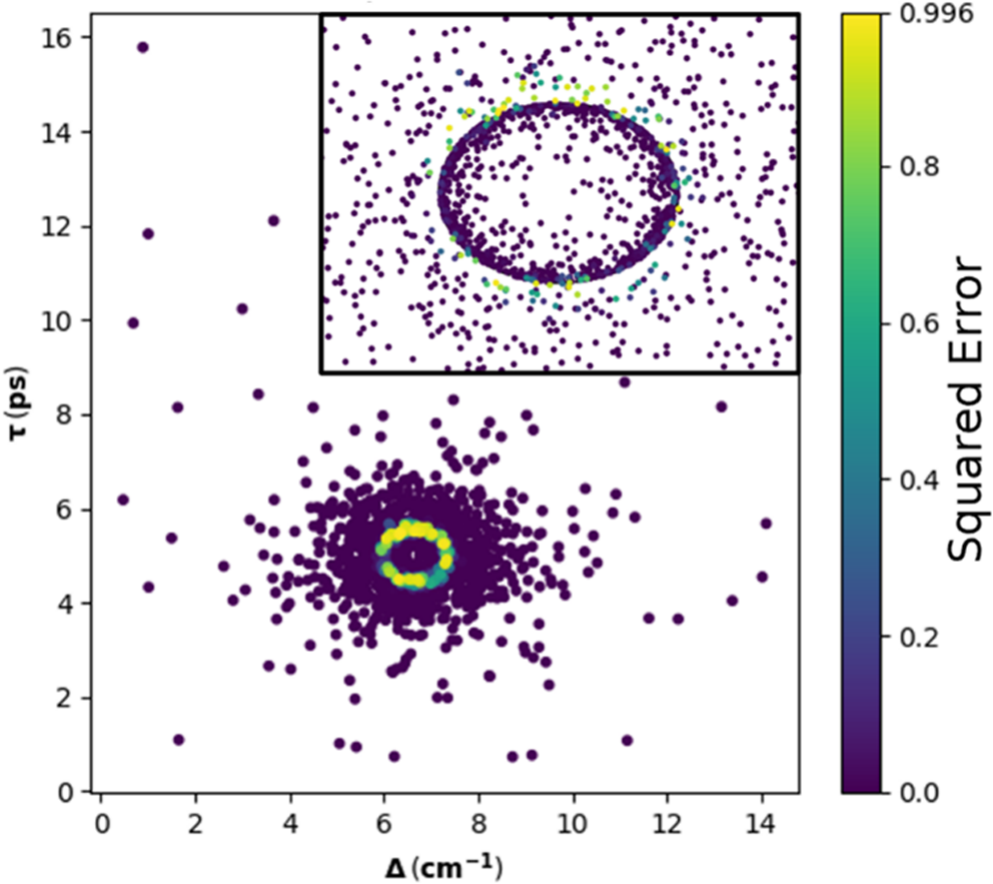
Squared-error of each validation sample from data set 1, comparing the output value of the network to the truth value of the sample. The inset is a zoomed-in display of the parameter space immediately around the classification boundary.

Considering only the model trained on a pump-time of 1 ps and pump–probe delay of 2.8 ps for data set 1, Figure 5 plots the squared error of each validation sample. We can see that the model is able to accurately classify all samples far away from the boundary (either inside or outside) and that those samples with the highest error are those closest to the classification boundary. This is expected, as the samples nearest the boundary should be the most difficult for the model to classify.

**Figure 6:**
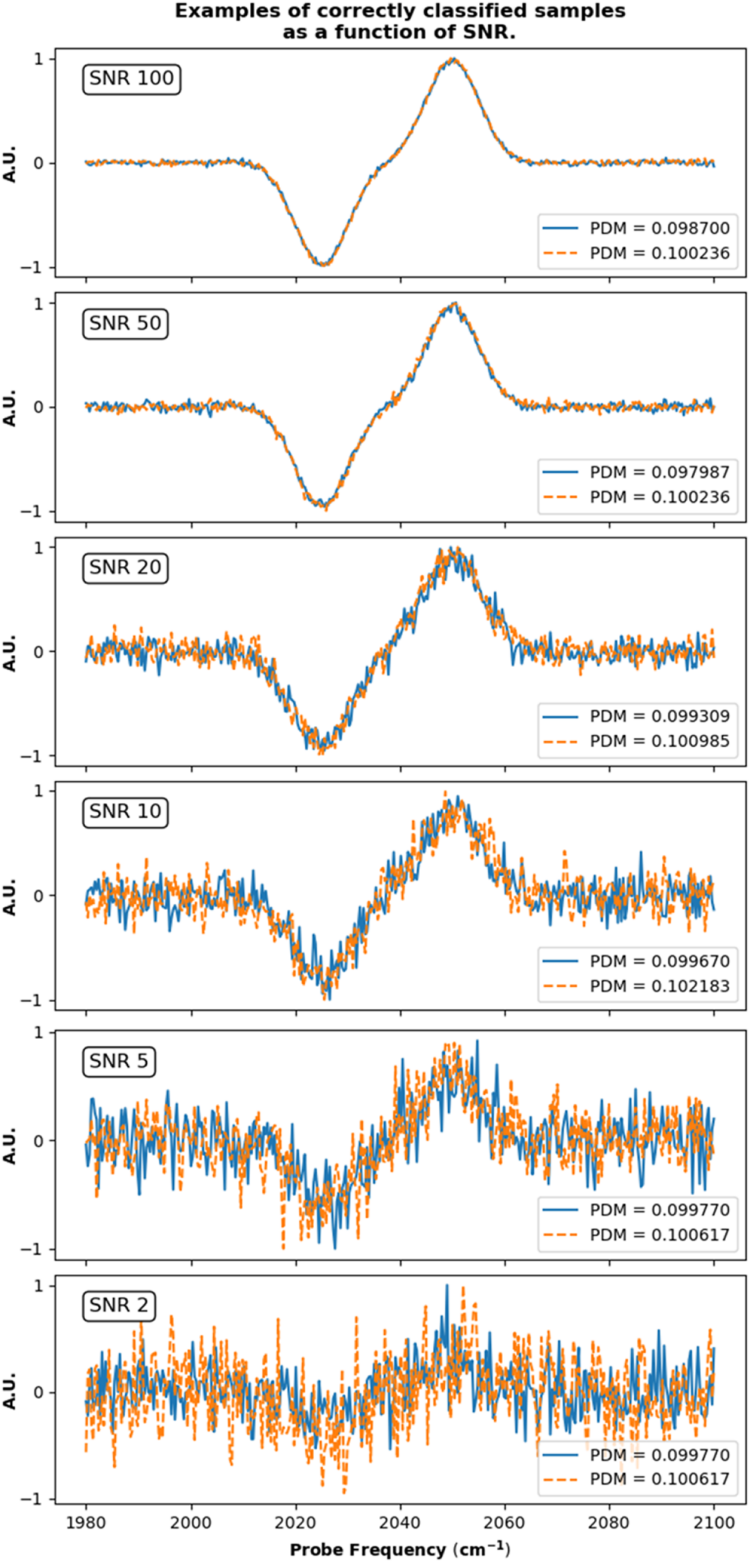
Comparison between samples that were correctly classified by the networks for each SNR. Each plot displays the real component of the complex data for two samples in each data set, where one sample is inside the classification boundary and the other is outside the classification boundary. An example of the samples’ location in Kubo parameter space is shown in Figure S8.

The discrimination of the models is impressive even for samples that have nearly identical Kubo parameters. For example, when again considering the results for the model trained on a pump-time of 1 ps and pump–probe delay of 2.8 ps for data set 1, the samples defined by the Kubo parameters (Δ, τ) = (6.257 cm^–1^, 5.386 ps) and (Δ, τ) = (6.244 cm^–1^, 5.388 ps) have PDMs of 0.09870 and 0.1002 respectively, i.e., the two samples are on either side of the classification boundary. Despite their small relative differences, however, the model is still able to classify each sample into the correct category. The actual data input to the network for each of the two samples is seen in the top panel of Figure 6. The lower panels of Figure 6 display other very similar samples that were correctly classified by the models despite decreasing the SNR in each case.

A substantial result here is that with a single slice of the entire 2D IR data set, i.e., a single waiting-time and a single value of the pump-delay, the spectral information in the slice is enough that a well-trained network can accurately classify exceptionally small differences in the Kubo parameters even with a SNR as low as 2, which could be captured experimentally in a few hundred laser shots, which could require less than a second of data acquisition time even for poor chromophores at low concentrations.

Realizing that the SNR itself might change which slice is optimal, we further test the networks by training only on data set 1 but with data of decreasing SNR. We find that as the SNR of the training data is lowered, the optimal pump/waiting-time slice trends toward lower values across both axes (Figure S9). This is not unexpected as the signal is naturally more intense in these regions. We observe that the BSS is approximately logarithmic with respect to the SNR, such that SNR values below 50 have a significant effect on the classification ability of the network. Thus, although it is possible for a network to accurately classify very similar data even at low SNR the network becomes less robust as the SNR decreases. Thus, the SNR remains a limiting factor for the ability of our networks to classify spectra. Adding a denoising operation to the spectra prior to input to the network could pair exceptionally well with our models, producing high classification skill even at low SNR.^25^

Since the training data was purposely biased to focus on the classification boundary, we also performe an analysis of a model trained on biased training data and subsequently tested on data uniformly distributed across the dataspace. We generate a model trained on SNR 100 data, where the training and validation sets utilize the same Cauchy distribution previously described to ensure many training samples are located near the classification boundary. We then test that trained model on one million samples distributed uniformly across the dataspace in order to test any bias in the model that could have been generated due to the biased training set. We find that the trained model does not display poor classification ability in dataspace regions where there were fewer training samples. This is illustrated in Figure S10, which plots the binned squared-error of samples across the dataspace and shows that even regions with no training samples display approximately zero error.

## 4 Conclusions

We investigate the efficacy of utilizing artificial neural networks to classify samples based on their 2D-IR spectra. On samples with experimental 2D-IR spectra, we determine that not only is it possible to classify 2D-IR spectra with artificial neural networks, but that high classification accuracy is still possible with reduced data sets and low signal-to-noise ratios. Using only the real-valued component of a single waiting-time still results in over 99% validation accuracy for data sets with SNR above three. On simulated 2D-IR spectra, we show that ANNs are capable of parsing 2D-IR spectra that display subtle and minute spectral differences and that classification is still possible with as little as one pump/waiting-time slice of the 2D-IR spectrum. Furthermore, we identify trends in terms of how the optimal pump/waiting-time slice relates to the reference Kubo parameters and the SNR of the spectra.

This work is limited to experimental spectra with relatively large differences and simulated spectra with relatively small differences but with a simple Kubo model. In future work, we plan to explore the capabilities of our ANN classification approach on experimental spectra that feature similar spectra and complicated line-shape models. This includes increasing the parameter space from two to 14 dimensions and features the complication of experimental fluctuations and errors.

## Supporting Information Available

Detailed solvent discrimination results; experimental spectral processing methods; simulated spectra processing methods; details of neural-network architecture; supplemental figures of simulated spectra classification. (PDF)
