# Supplementary material for: Classification of Samples via Neural-Network Augmented Two-Dimensional Infrared Spectroscopy
Source: J Phys Chem B. 2025 May 1;129(19):4738–46. doi: 10.1021/acs.jpcb.4c08573 (PMC12086836; doi:10.1021/acs.jpcb.4c08573)
Supplement: Supplementary file 1 — jp4c08573_si_001.pdf [file jp4c08573_si_001.pdf]

Classification of Samples via Neural-Network Augmented Two-Dimensional Infrared Spectroscopy

Evan B. Schroeder and Christopher M. Cheatum*

Department of Chemistry, University of Iowa, Iowa City, Iowa 52242, USA

*Author to whom correspondence should be sent. E-mail: christopher-cheatum@uiowa.edu

Supporting Information

Table S1 Loss of each solvent from Section 2 as a function of SNR.

Mean Loss x 10^{-5}

SNR	BMIM-TFSI	DMF	DMSO	Glycerol	H2O
**100**	0.17	1.57	2.89	0.75	0.10
**50**	0.06	3.48	4.93	1.20	0.40
**20**	0.26	4.70	11.84	4.54	5.21
**10**	0.40	39.42	22.73	5.64	8.79
**5**	1.07	62.56	901.07	0.68	5.41
**2**	2256	151152	39202	56.31	513.5
**1**	184921	17717	259573	223965	9682

Table S2 The percent difference magnitude of each solvent compared to H_2O.

PDM Compared to Water

BMIM-TFSI	DMF	DMSO	Glycerol
3881%	1344%	293%	6984%

Section S1: *Time Required to Collect 2D IR Datasets*

Consider a recent analysis of the enzyme formate dehydrogenase by the Cheatum group.[1] Each pump time (t_{pump}) required eight laser shots, and each 2D spectrum for a single waiting-time (T_w) required 167 pump times. Each 2D spectrum was averaged-in about 100 times, there were 101 different waiting-times collected, and 10 replicates of the spectra were collected.

$$\left(8\ \frac{laser\ shots}{t_{pump}}\right) \times \left(167\ \frac{t_{pump}}{spectrum}\right) \times \left(100\ \frac{spectra}{T_w}\right) \times (101\ T_w) \times (10\ replicates) = 134{,}936{,}000 \text{ laser shots} \quad \text{(S1)}$$

Thankfully, the repetition rate of the laser was 2 kHz, resulting in a total time to collect the 2D IR spectrum for a single sample of about 19 hours.

$(134{,}936{,}000 \text{ laser shots}) \div \left(2000 \frac{\text{shots}}{s}\right) = 64{,}468\ s \approx 18.7\ hrs$	(S2)

In the case of undersampling the waiting-time axis by only collecting two waiting-times, as discussed in the introduction, the collection time per sample is slightly over twenty-two minutes.

Section S2: *Normalization of Experimental Spectra*

First, all spectra were cropped to the frequency range $2050\ cm^{-1} - 2250\ cm^{-1}$ along the pump and probe axes. This initial crop was done to remove the high-noise pixels that are present on the edges of the spectra.

Convolutional neural networks are traditionally considered to be translationally invariant. That is, a convolutional neural network should be able to produce the classification result regardless of where the feature of interest is located within the input image. However, in general that concept is not true.[2, 3] As such, the spectra were manipulated such that the 0→1 vibrational transition peak was at a constant pixel coordinate for each spectrum.

Next, each spectrum was cropped to a global minimum and maximum frequency along each axis. These global values were determined by finding the smallest difference between the 0→1 peak and the edge of the spectrum. For example, to find the global maximum frequency along the pump axis, the differences between a spectrum's 0→1 peak and its maximum pump value were calculated for all samples and the smallest value chosen as the global maximum.

After cropping, each spectrum was normalized to lie within the intensity range [-1, 1]. Experimental spectra may have higher or lower signal depending upon the sample composition, such as chromophore concentration or solvent. Therefore, the normalization is necessary to ensure the neural network does not learn to categorize samples based upon absolute signal. However, relative signal within a single sample, such as from one waiting-time to another, is preserved as that information could be useful for classification.

Finally, each spectrum was interpolated such that all spectra contained the same pixel density. This global pixel density was set by the smallest pixel density of all sample spectra. The final spectra had 227 rows and 59 columns, i.e. the pump axis was 59 pixels in length and the probe axis was 227 pixels in length.

Following cropping and interpolation, Gaussian noise was added to each spectrum such that each spectrum had a desired SNR.

Thus, after data transformations, each sample spectrum had identical 0→1 peak pixel coordinates, frequency ranges, pixel densities, maximum absolute intensities, and signal-to-noise ratio. An example of the full data transformation process can be seen in Figure S2.

Section S3: *Noise Analysis of Experimental Spectra*

SNR was determined for each sample as the difference in spectrum maximum and minimum, divided by the root-mean-square noise, as in Equation S3.

$$SNR = \frac{\max(spectrum) - \min(spectrum)}{RMS(noise)} \tag{S3}$$

The RMS noise was calculated based on the pixels in range $[\omega_1(px), \omega_3(px)] = [:25, 165:]$, i.e. the top left of the contour-map. This region was chosen as it contained minimum contribution from the vibrational transition peaks.

To generate a spectrum with the desired SNR, scaled Gaussian noise was added to each spectrum, after which the spectrum was re-normalized to the interval [-1, 1]. This scaling takes the form of Equation S4, where μ is the scaling factor for the generated noise and $px_{original}$ is the intensity of a noisy pixel prior to addition of generated noise.

$$SNR_{final} = \frac{signal_{original}}{\sqrt{\frac{1}{n}\sum_i^n \left[\left(px_{original}\right)_i + \mu \times (px_{noise})_i\right]^2}} \tag{S4}$$

Rearranging terms leads to Equation S5.

$$0 = \mu^2 \sum_i^n (px_{noise})_i^2 + \mu \sum_i^n 2\left(\left(px_{original}\right)_i \times (px_{noise})_i\right) + \sum_i^n \left(px_{original}\right)_i^2 - n\left(\frac{signal_{original}}{SNR_{final}}\right)^2 \tag{S5}$$

The appropriate scaling factor can then be calculated via the quadratic equation as in Equation S6.

$$\mu = \frac{-b \pm \sqrt{b^2 - 4\,a\,c}}{2\,a} \tag{S6}$$

Where $a = \sum_i^n (px_{noise})_i^2$, $b = \sum_i^n 2\left(\left(px_{original}\right)_i \times (px_{noise})_i\right)$, and $c = \sum_i^n \left(px_{original}\right)_i^2 - n\left(\frac{signal_{original}}{SNR_{final}}\right)^2$. Note that using either the positive or negative result from the quadratic equation results in the same resulting SNR. An example of the addition of scaled generated noise to the 2D IR spectra can be seen in Figure S1.

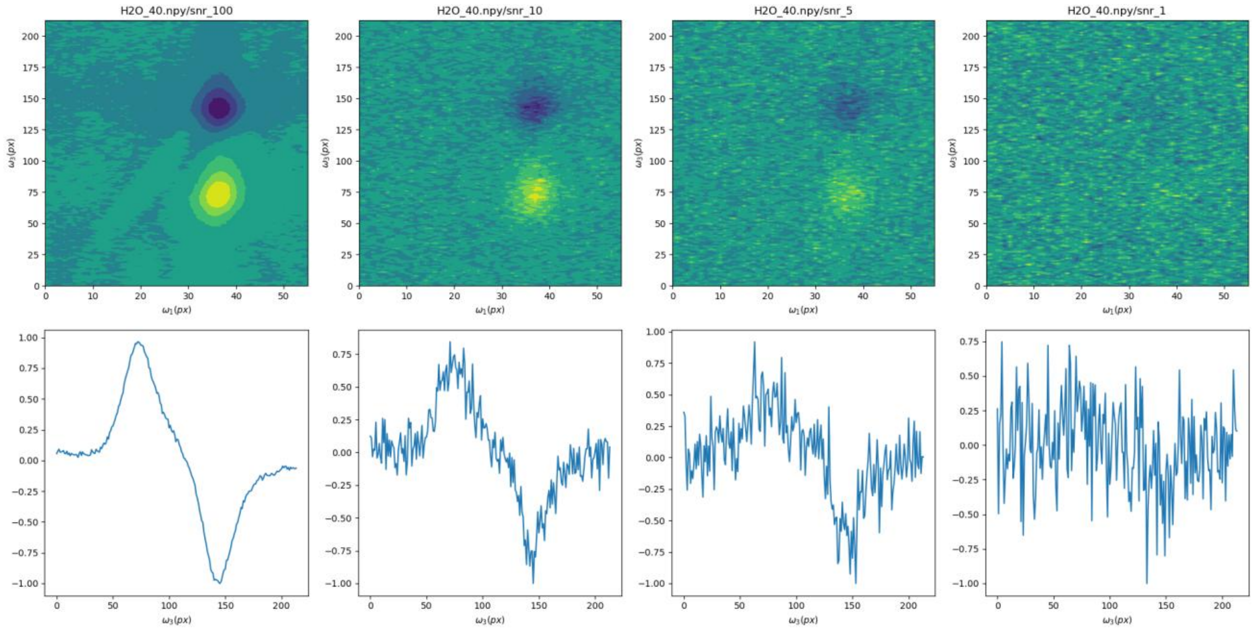

Figure S1 Examples of various SNR. Each column is of the same original spectrum, but with a different scaling factor used for the generated noise. The bottom row displays the vertical slices though the center of the peaks for each spectrum.

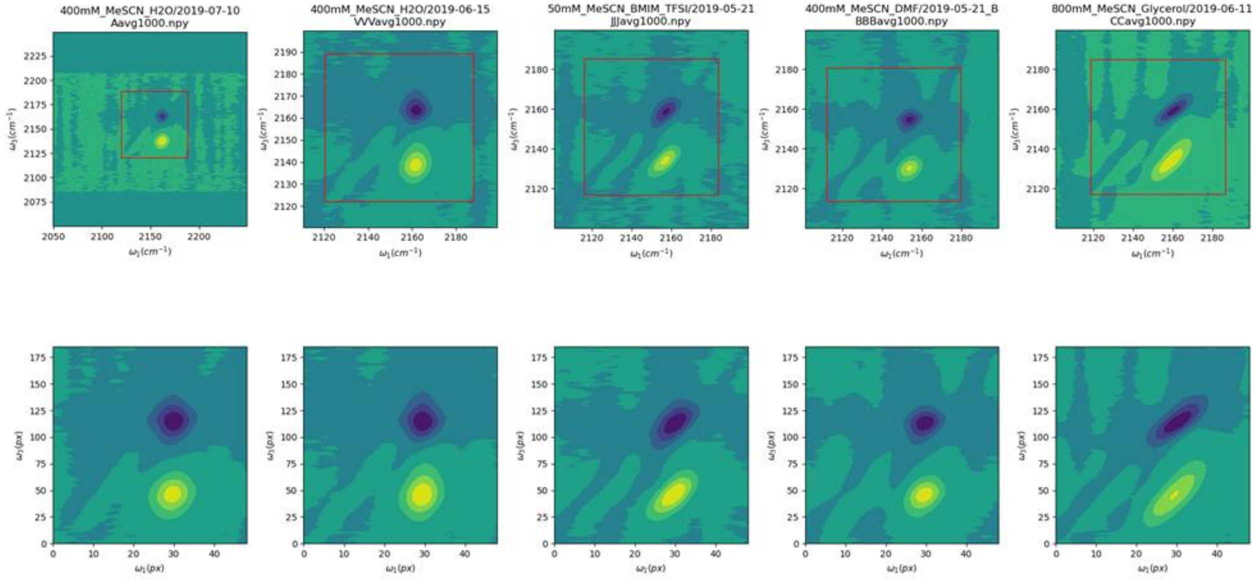

Figure S2 An example of the data transformation process. The top row displays example spectra of five samples, with the red square indicating the crop-range. The bottom row displays the resulting spectra after cropping, translation, normalization, and interpolation. Note that the top row has axis units of frequency, while the bottom row has units of pixels.

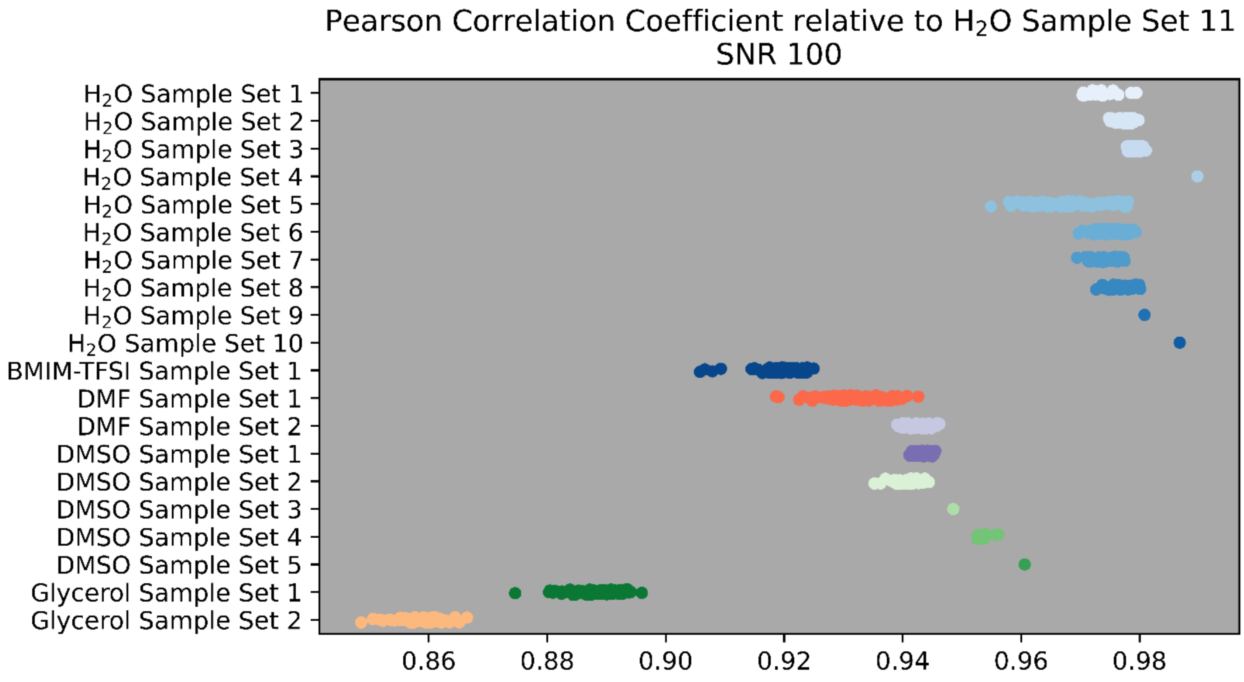

Figure S3 The Pearson Correlation Coefficient (PCC) of each spectrum relative to a single water solvent sample spectrum at SNR 100.

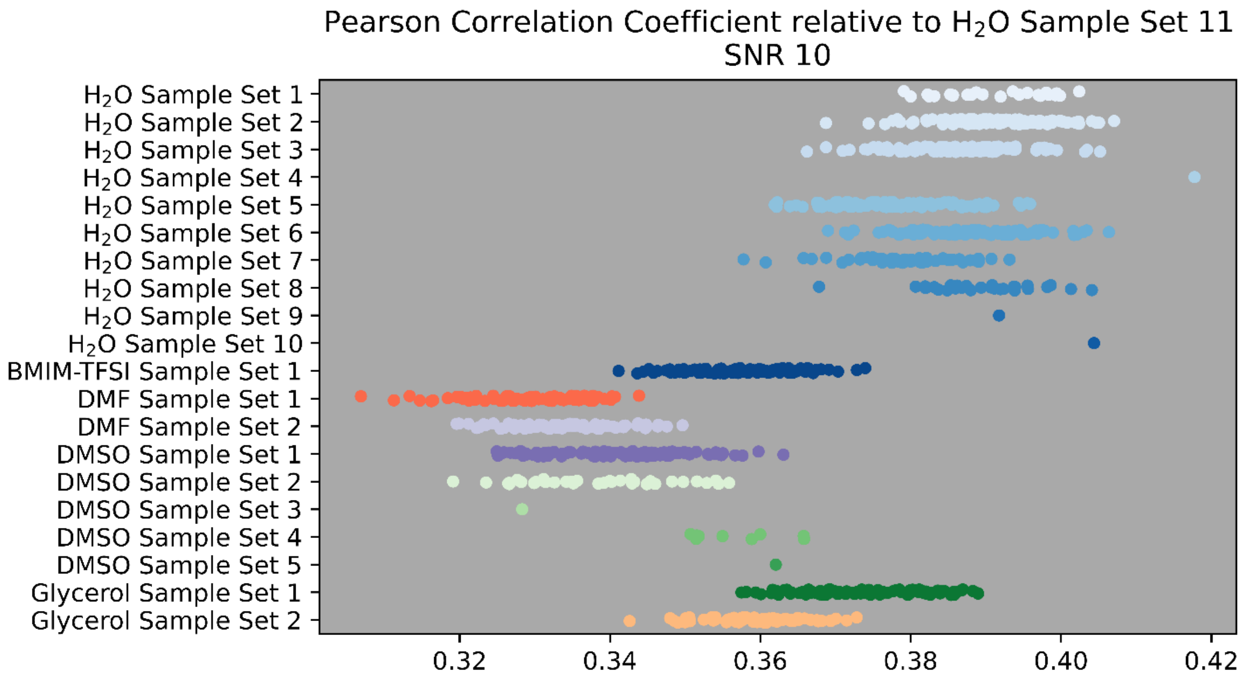

Figure S4 The Pearson Correlation Coefficient (PCC) of each spectrum relative to a single water solvent sample spectrum at SNR 10.

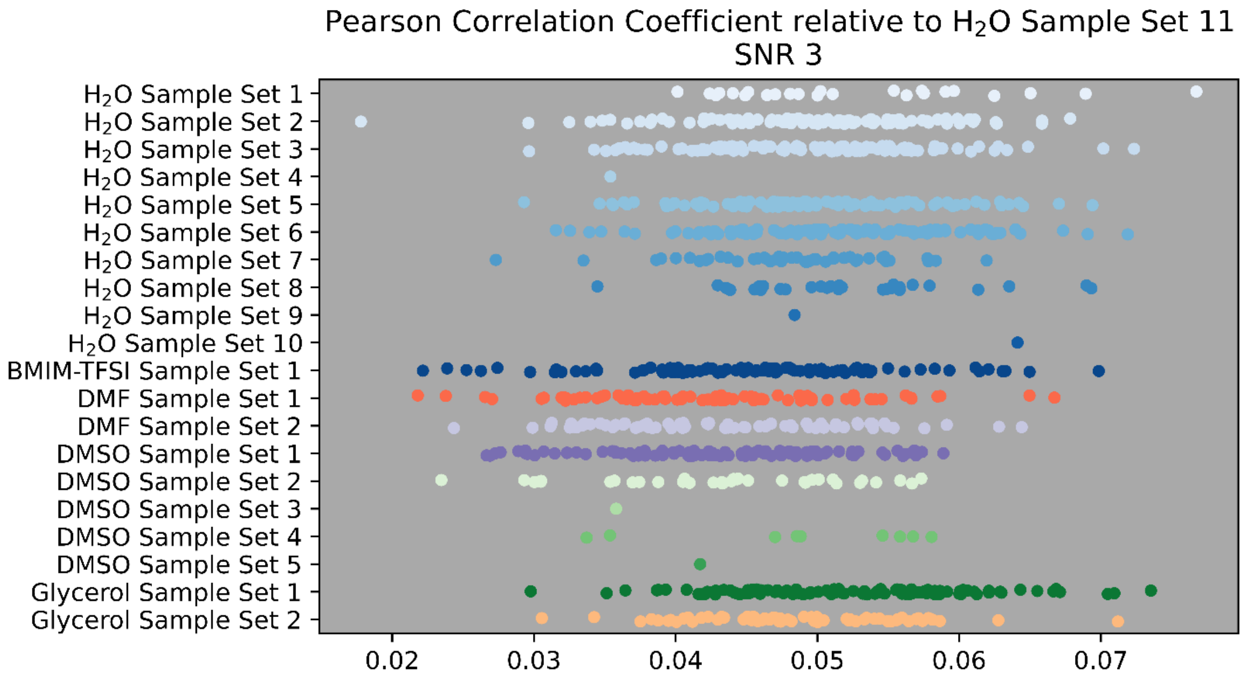

Figure S5 The Pearson Correlation Coefficient (PCC) of each spectrum relative to a single water solvent sample spectrum at SNR 3.

Section S4: *Effects of Repeated Spectra*

To obtain a balanced dataset, in the experimental sample section we generated 54 repeats of existing water spectra. These repeats were randomly distributed among the k-folds and thus could have an effect on the results by biasing the accuracy to higher values. To test this, we generated two datasets: one in which the repeats were grouped within a single k-fold and one in which they were distributed equally among the k-folds. Models trained on the two datasets at SNR 2 displayed validation accuracies of $83.3\% \pm 4.1\%$ for the grouped dataset and $88.6\% \pm 2.5\%$ for the distributed dataset. We therefore conclude that the distribution of repeated spectra has a minimal impact on our results.

Section S5: *Simulated Spectra Generation*

In this work, the spectral parameters used were:

$(\boldsymbol{\omega_{01}})$ **:** In units of wavenumbers (cm^{-1}), the 0→1 transition center frequency.

$(\boldsymbol{\delta\omega})$ **:** In units of wavenumbers (cm^{-1}), the calibration mismatch error between the pump and probe axes.

$(\boldsymbol{\omega_{Anh}})$ **:** In units of wavenumbers (cm^{-1}), the anharmonicity of the 0→1 and 1→2 peaks.

$(\boldsymbol{\omega_{s}})$ **:** In units of wavenumbers (cm^{-1}), the frequency shift of the pump axis when collected in the rotating frame.

$(\boldsymbol{\beta})$ **:** Unitless, a scaling factor for the Kubo amplitude of the 1→2 transition relative to the 0→1 transition.

$(\boldsymbol{\tau})$ **:** In units of picoseconds, the time-constant of the Kubo component.

$(\boldsymbol{\Delta})$ **:** In units of wavenumbers (cm^{-1}), the amplitude of the Kubo component.

Of these, only the Kubo time-constant and amplitude were varied between simulated samples, with all other parameters remaining constant. The Kubo contribution to the lineshape is given by Equation S7, for pump axis t_1, probe axis t_3, and waiting-time axis T_w.

$$G_{Kubo} = \exp\left(-(2\pi c)^2\,\Delta^2\,\tau^2\left(\mathrm{expm1}\left(\frac{-t_1}{\tau}\right)+\frac{t_1}{\tau}\right)\right) \times \exp\left(-(2\pi c)^2\,\Delta^2\,\tau^2\,\beta^2\left(\mathrm{expm1}\left(\frac{-t_3}{\tau}\right)+\frac{t_3}{\tau}\right)\right) \times \exp\left(-\eta\,(2\pi c)^2\,\Delta^2\,\tau^2\,\beta\exp\left(\frac{-T_w}{\tau}\right)\mathrm{expm1}\left(\frac{-t_1}{\tau}\right)\,\mathrm{expm1}\left(\frac{-t_3}{\tau}\right)\right)$$	(S7)

The ground state bleach and excited state absorption contributions to the response functions are given in Equations S8 and S9, where η equals -1 for the rephasing response and $+1$ for the non-rephasing response, R_- and R_+ respectively in Equation S10.

$$GSB = A_{01}\exp\Big(i\,2\pi c\,\big(t_3(\omega_{01}-\omega_0)+\eta\,t_1(\omega_{01}-\omega_s+\delta\omega)\big)\Big)\times G_{Kubo}$$	(S8)

$$ESA = A_{12}\exp\Big(i\,2\pi c\,\big(t_3(\omega_{01}-\omega_{Anh}-\omega_0)+\eta\,t_1(\omega_{01}-\omega_s+\delta\omega)\big)\Big)\times G_{Kubo}$$	(S9)

$$R_{\pm} = ESA - GSB$$	(S10)

The final 2D IR spectrum is calculated via Equation S11, where $\mathcal{F}\{R_{\pm}(t_3)\}$ represents the Fourier-transform across the probe axis of the Rephasing and Non-Rephasing response functions, and $*$ represents the complex conjugate.

$$spectrum = \mathcal{F}\{R_+(t_3)\} + \mathcal{F}\{R_-(t_3)\}^*$$	(S11)

The SNR was adjusted by adding Gaussian noise to the spectra. In this case, signal was defined as the absolute maximum of the transient absorption spectrum, which is intensity vs. probe frequency at pump time zero and waiting time zero ($t_1 = 0\,ps, T_W = 0\,ps$). Gaussian noise was generated, then scaled according to the desired SNR as in Equation S12.

$$SNR = \frac{S}{\xi \times RMS}$$	(S12)

The summation of two noisy signals yields a root-mean-squared (RMS) value that is the root of the sum of squares of the signals' RMS values $\left(RMS_{Total} = \sqrt{RMS_1^2 + RMS_2^2}\right)$. However, since the simulated spectra are considered to have zero noise, the summation of the simulated

spectra and the scaled noise results in a noisy signal with RMS equal to the scaled noise. Thus, the proper scaling factor can be determined via Equation S13.

$$\xi = \frac{S}{SNR \times RMS} \quad (S13)$$

Spectra were then normalized such that all values were on the interval [-1, 1] real and [-1i, 1i] imaginary. In practice, the scaling was most often determined by the real part of the complex spectrum. Relative scaling between waiting times was preserved, as it is concentration independent and is information the neural network could use for categorization.

All spectra simulation was done in Python using a custom simulation class, utilizing NumPy for matrix manipulation.[4] To help prevent float overflow errors, all calculations were done with the "longdouble" datatype from NumPy, which evaluates as a 128-bit float provided it is supported by the platform. For example, on 64-bit Windows builds, "longdouble" will evaluate as a 64-bit float, instead of the full 128-bits.

Section S6: *Neural Network Architecture*

The three-dimensional ResNet used for categorization of simulated spectra expects inputs of shape (B, C, F, H, W), where B is the batch-size, C is the number of channels, F is the number of frames, H is the frame height, and W is the frame width. The network used the default hyperparameters (i.e., those parameters that are chosen by the user prior to training) from the PytorchVideo repository, save the number of input channels (2), the number of model classes (1), the learning rate $(1e^{-4})$, and the pooling kernel size (1, 7, 1).[5]

All training and validation was done with Pytorch, utilizing Pytorch-Lightning as a wrapper.[6, 7] Tuning, training, and validation were performed on the University of Iowa Argon High-Performance Computing system, utilizing a mixture of NVIDIA GPUs. For each training session, the simulated spectra were held in memory instead of being saved to the hard disk. This significantly speeds up training, provided the datasets are not too large compared to the memory capacity of the compute node.

To choose samples within the miss region, we considered the classification boundary as the surface of an n-sphere of two dimensions. By selecting random points uniformly on the n-sphere and then scaling the vector by a random amount, we could select random samples within the classification boundary.

To uniformly select points on the n-sphere, we utilized the method by Marsaglia.[8] For each sample, we first selected two random values from a Normal distribution (one for Δ and one for τ), then divided each value by the norm of the two. This produced a vector $\langle \mathrm{d}, t \rangle$ which lies on the surface of the unit n-sphere. For the scaling value, we first selected a random value (η) from the Cauchy distribution cropped from zero to one, centered at one, and with a half-width at half-

maximum equal to the PDM classification boundary. The ⟨d, t⟩ vector selected in the previous step was then scaled according to Equation S14.

$\vec{v} = \langle d, t \rangle \times \eta^{\frac{1}{2}}$	(S14)

Finally, the scaled vector was then translated such that the apo parameter coordinates defined the origin, and scaled such that the classification boundary around the apo coordinates defined the surface of the n-sphere, as in Equation S15. For this chapter, 0.1 was the PDM of the classification boundary.

$\Delta_s = \Delta_{apo}(d \times PDM + 1), \quad \tau_s = \tau_{apo}(t \times PDM + 1)$	(S15)

Choosing samples from the hit distribution is done in much the same way, with additional considerations. The initial ⟨d, t⟩ vector is still selected uniformly on the two-dimensional n-sphere, but the scaling parameter is instead sampled from a Cauchy distribution, centered at one, for all values greater than one. Scaling the vector, then translating and scaling the result is done according to Equations 3.9 and 3.10. However, an additional check must be done to ensure Δ_s and τ_s are not so large as to create an overflow error in the calculation. The maximum value of a 128-bit float is $\sim 10^{4932}$, which can sometimes be violated by the final term of Equation S7. Therefore, the check in Equation S16 is done for each (Δ_s, τ_s) pair in the hit distribution to prevent overflow errors.

$\frac{(2\pi c)^2\, \Delta^2\, \tau^2\, \beta\, \text{expm1}\left(\frac{-t_1}{\tau_c}\right)\, \text{expm1}\left(\frac{-t_3}{\tau_c}\right) \exp\left(-\frac{T_w}{\tau_c}\right)}{\log 10} < 4932$	(S16)

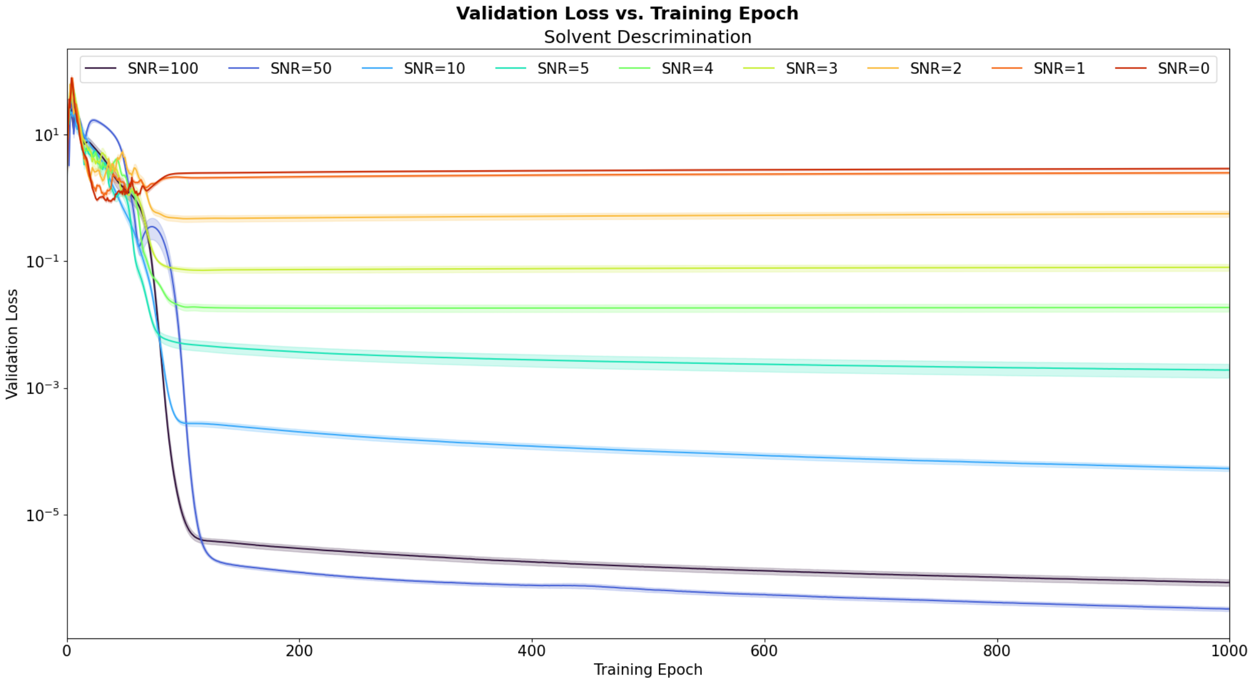

Figure S6 Validation loss as a function of training epoch. Bands represent the SEM of the 5 k-folds.

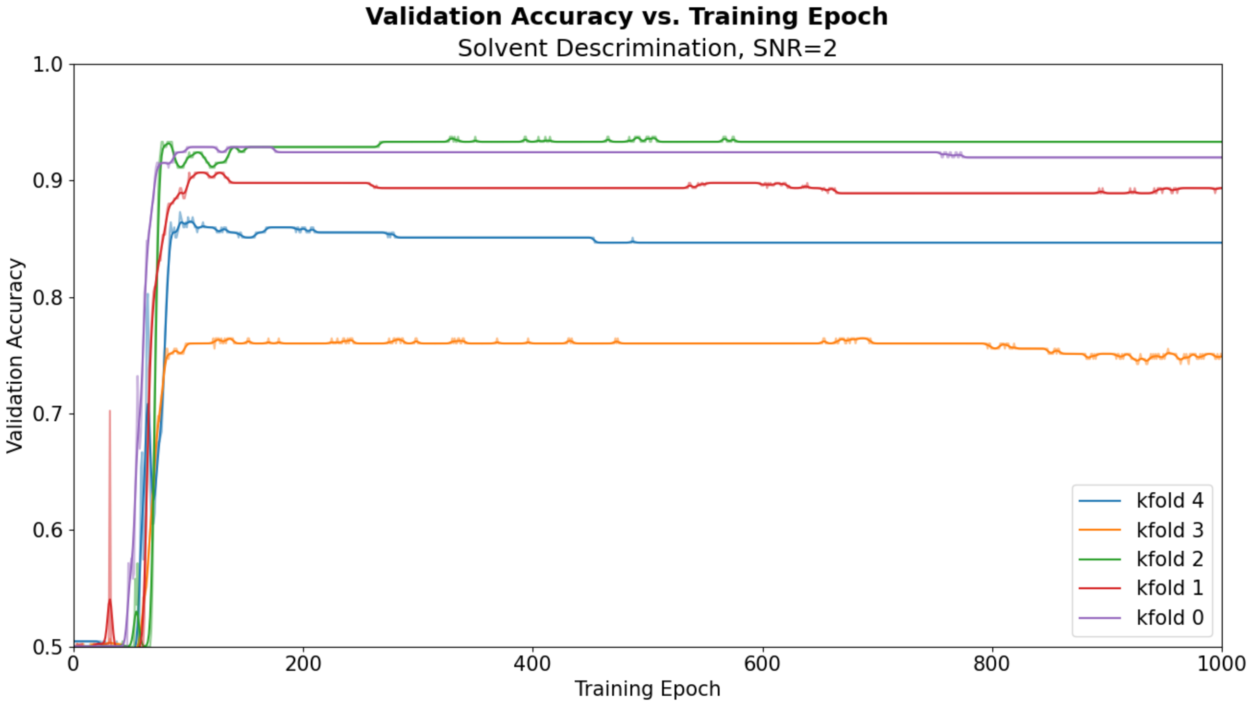

Figure S7 Validation accuracy of the model trained on experimental data as a function of the training epoch.

Table S3 For each dataset, the pump / waiting-time coordinates that produced the best validation BSS. The validation accuracy corresponds to the accuracy of the validation set at the training epoch that resulted in the best BSS.

**Dataset**	**Δ (cm^{-1})**	**τ (ps)**	**Pump (ps)**	**Tw (ps)**	**Val BSS**	**Val Acc.**
**1**	6.667	5	1	2.8	0.852	0.950
**2**	13.333	5	0.384	5.4	0.886	0.963
**3**	6.667	10	1	4.8	0.847	0.946
**4**	13.333	10	0.384	8.6	0.895	0.964
**5**	13.333	2.5	0.384	3.2	0.870	0.954
**6**	3.333	10	1.92	5.4	0.826	0.940
**7**	3.333	5	1.536	4.8	0.842	0.946
**8**	6.667	2.5	1	1.2	0.841	0.946
**9**	3.333	2.5	1.92	2.4	0.827	0.940
**10**	6.667	1.25	0.96	1.2	0.835	0.943
**11**	13.333	1.25	0.384	1.2	0.872	0.956
**12**	3.333	1.25	2	1.2	0.748	0.911
**13**	6.667	0.75	1	0.4	0.810	0.932
**14**	13.333	0.75	0.576	0.8	0.849	0.948
**15**	3.333	0.75	2	0	0.603	0.850

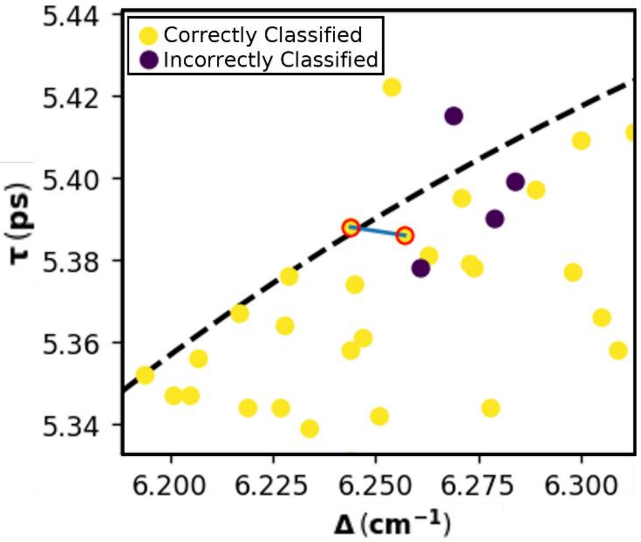

Figure S8 The location in Kubo parameter space of the two samples displayed in the upper panel of Figure 6. The two samples are denoted with red circles, connected by a blue line.

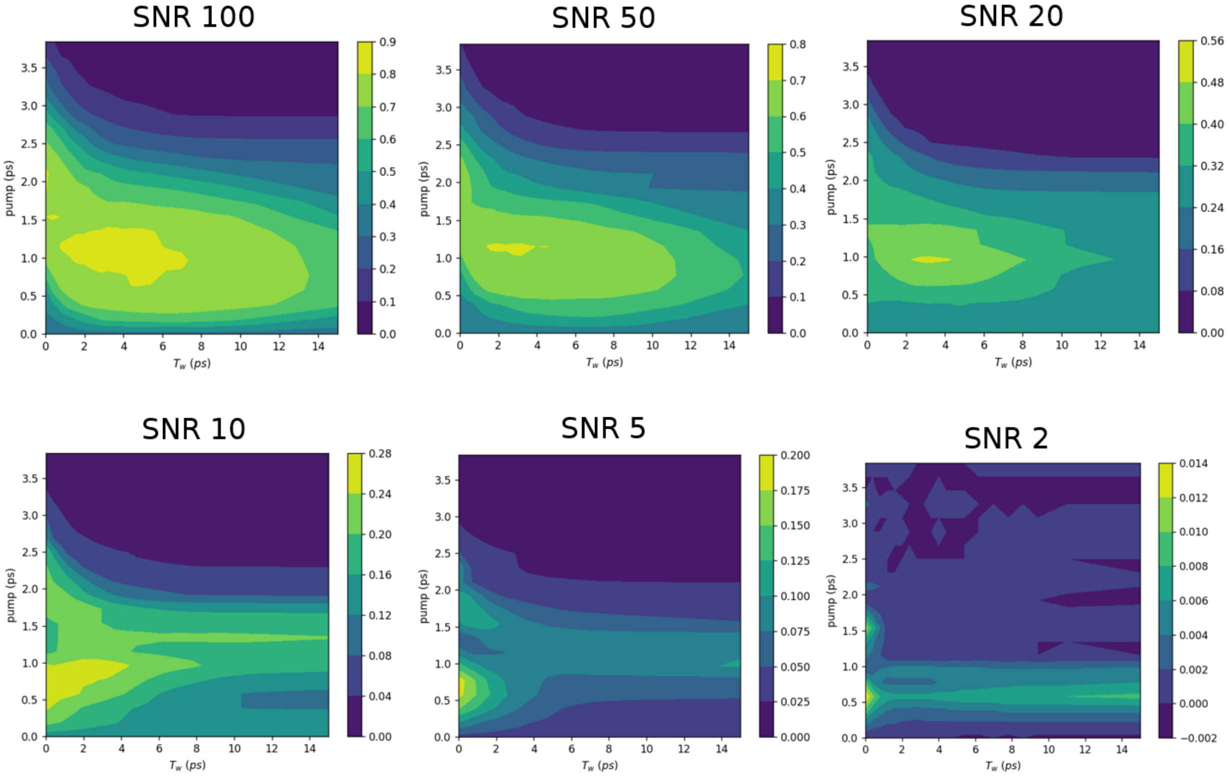

Figure S9 The best BSS achieved as a function of the pump time and waiting-time, as was done in Figure 4 of the main text. These plots display the results for a single dataset, but with decreasing SNR.

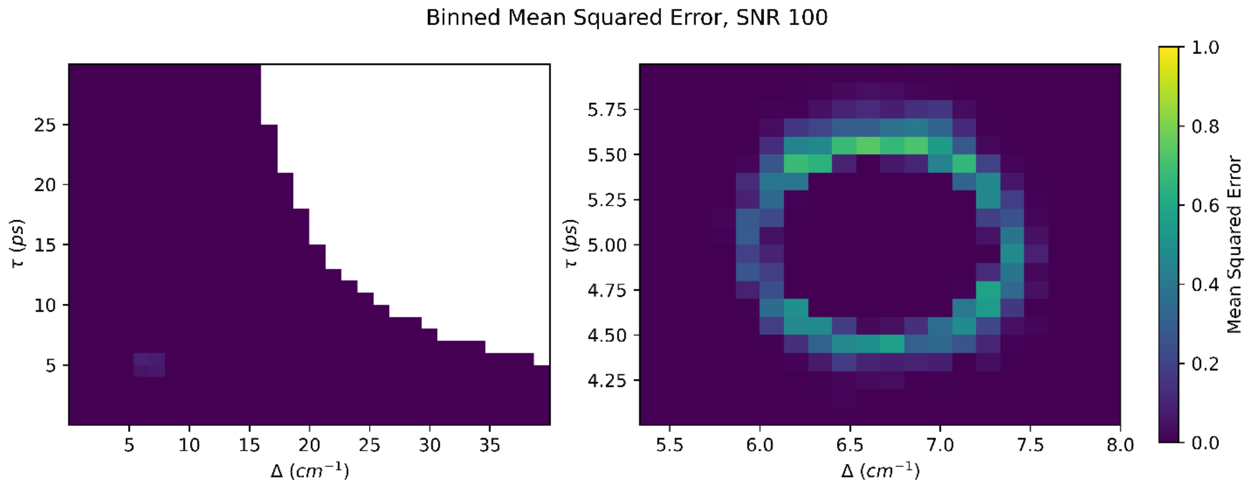

Figure S10 The binned squared-error of samples distributed uniformly across the dataspace, from a model trained data biased to focus on the classification boundary. Note that regions far away from the boundary, which contain almost no training samples, still display approximately zero error.

References

1. Pagano, P., et al., *Oscillatory Active-Site Motions Correlate with Kinetic Isotope Effects in Formate Dehydrogenase.* Acs Catalysis, 2019. **9**(12): p. 11199-11206.
2. Biscione, V. and J.S. Bowers, *Convolutional Neural Networks Are Not Invariant to Translation, but They Can Learn to Be.* 2021.
3. Biscione, V. and J.S. Bowers, *Learning online visual invariances for novel objects via supervised and self-supervised training.* Neural Networks, 2022. **150**: p. 222-236.
4. Harris, C.R., et al., *Array programming with NumPy.* Nature, 2020. **585**(7825): p. 357-362.
5. Fan, H., et al. *PyTorchVideo: A deep learning library for video understanding*. in *Proceedings of the 29th ACM international conference on multimedia*. 2021.
6. Falcon, W., *PyTorch Lightning*. 2019.
7. Paszke, A., et al., *Pytorch: An imperative style, high-performance deep learning library.* Advances in neural information processing systems, 2019. **32**.
8. Marsaglia, G., *Choosing a Point from the Surface of a Sphere.* The Annals of Mathematical Statistics, 1972. **43**(2): p. 645-646, 2.
